# Socio-demographic, maternal, and infant characteristics associated with early childhood development delays among children of young mothers in Brasília, Brazil

**DOI:** 10.1371/journal.pone.0266018

**Published:** 2022-03-30

**Authors:** Lily Kofke, Rafael Pérez-Escamilla, Muriel Bauermann Gubert, Gabriela Buccini

**Affiliations:** 1 Department of Social and Behavioral Sciences, Yale School of Public Health, New Haven, Connecticut, United States of America; 2 Department of Nutrition, University of Brasília, Brasília, Federal District, Brazil; 3 Department of Social and Behavioral Health, University of Nevada Las Vegas School of Public Health, Las Vegas, Nevada, United States of America; School of Public Health, University of São Paulo, BRAZIL

## Abstract

**Background:**

Holistic attention to adolescent health is needed to sustain the benefits of investment in early childhood development. Any such interventions must make sure to address the needs of adolescent and young adult parents. This study explored the social and demographic maternal variables associated with risk of early childhood development (ECD) delay for children of young mothers in Brazil.

**Methods:**

Cross-sectional secondary data analysis was done using data from young mothers (aged 13–24) and their children (aged 0–2), collected from community health centers in Brasília, Brazil, between 2017–2018. The Denver Developmental Screening Test II was used to assess risk of ECD delay outcomes. Descriptive analyses were conducted across the full sample and sub-groups of adolescent (13–19) and young adult (20–24) mothers. Multivariable logistic regressions based on theory modelling approach were conducted for the full sample to examine the associations between maternal age and risk of ECD delay, adjusted for a battery of household, maternal, pregnancy, and infant variables.

**Results:**

Risk of ECD delay was found in 17.39% (N = 76) of the children who participated (N = 437). No significant differences in risk of ECD delay were found for children of adolescent mothers compared to children of young adult mothers. Across the full sample, 60.36% (N = 236) of mothers were living in poverty, 73.17% (N = 319) had 9 or more years of education, and 86.14% (N = 373) were not working outside the home at time of data collection. Furthermore, 90.11% (N = 392) did not identify as head of their household and 73.68% (N = 322) were primiparous. Socially-mediated factors such as lower maternal educational attainment, unemployment, and lack of household support were associated with increased risk of ECD delays for children under age 2. Adjusted logistic regression identified multiparity as an independent maternal factor associated with increased risk of ECD delay (AOR = 2.51; 95% CI, 1.23–5.13).

**Conclusions:**

Multiparity was the only independent maternal factor associated with ECD delay among children under 2 years old. Other socio-demographic factors relevant to young mothers may influence ECD delays. Ensuring sustained, concurrent attention to children’s and young parent’s developmental needs may improve multi-generational health outcomes.

## Introduction

An estimated 10 million unintended pregnancies among women age 15–19 occur globally each year [1, 2]. Adolescent and young adult mothers are, by definition, experiencing a period of development shaped by shifts in biology and social-roles, during which their behaviors can shape trajectories for their own health and their child’s health [3]. There is growing understanding that promoting health for future generations begins with ensuring adolescent and young adult health prior to and during pregnancy, benefitting intergenerational health and wellbeing [4]. Motherhood at an early age can often stem from interrelated structural social inequities, including poverty, gender biases, and violence [5]. These socio-demographic factors can define the expectations, norms, and experiences regarding transitions to adulthood such as parenting [6]. Subsequently, multisectoral policies and interventions targeting various risk factors, including access to education, employment, social support, and health care, are needed to support adolescent and young adult parents and their children to combat global-scale inequities [7–9], and to sustain the benefits of investments in early childhood development (ECD) [10].

Brazil is South America’s largest country with more than 212 million people, almost one-quarter of whom live in poverty and 6.5% of whom have monthly incomes below the poverty line [11]. Racial disparities have been documented in regard to socio-economic status, housing conditions, educational attainment, life expectancy, and prevalence of premature birth [12–14]. For women of reproductive age in Brazil, the intersectional relationships between race, class, education, and gender present unique social contexts that alter opportunities, stressors, and protective variables impacting maternal and child health [15]. Across all regions of Brazil, births among women age 15–19 have decreased in the past decade; fertility rates among those aged 10–14 have decreased everywhere except in the Northern and Northeastern regions [16], where income, education levels, and health indicators are lower compared to other regions of the country [17]. Similarly, adolescent pregnancy rates remain high in rural areas, where up to 18% of women have an unmet need for contraceptives [18]. Approximately 1 in 5 Brazilian women has their first child before age 20 and the country’s adolescent fertility rate of 68.4 births per 1,000 adolescents is above the Latin America and Caribbean average of 65.5 and the global average of 46.0 [17]. Trends in decreasing overall fertility but increasing adolescent fertility are seen in other Latin American countries and perpetuate inequalities related to lower school completion rates due to pregnancy and the subsequent health and social risks for mothers and their children [19].

Parenting home-visiting programs can be effective at connecting vulnerable young families to care and services, and in supporting caregivers in creating nurturing-care environments for their children. A recent evaluation of the Primeiros Laços (‘First Ties’) home visiting program for pregnant adolescents in São Paulo, Brazil, found that the program improved mothers’ wellbeing and parenting abilities [8]. These findings support existing evidence that adolescent parenting programs are associated with various positive benefits, including gains in maternal confidence, increased parenting skills and knowledge, and reduced risk of child abuse [20]. While parent home-visiting programs targeting young families have been found to help mitigate parents’ stress levels and improve their educational attainment and sexual health [21, 22], more research is needed regarding the particular developmental areas in which children of adolescent and young adult parents are at greatest risk. Additionally, it is unknown the extent to which parenting skill-building programs must be adapted to be responsive to the needs of the adolescent and young adult parents they serve [8].

Therefore, examining the socio-demographic, maternal, and infant factors and contexts within which adolescents and young adults become parents can help identify potential approaches to effectively mitigate threats to maternal health and ECD. Whether early pregnancy is a personal decision or the result of deficient public policies and programs, there is a need to better understand the factors associated with ECD delays in children of young and adolescent parents in Brazil to improve multigenerational health and wellbeing.

Our study aimed to identify maternal socio-demographic factors (e.g., educational attainment, employment, household support, parity) associated with an increased risk of ECD delays for children of adolescent and young adult mothers in Brazil.

## Materials and methods

### Study setting

Brazil’s capital, Brasília, also known as the Federal District, is located in the country’s Central-West region and is home to 3.1 million people [23]. In 2019, 57.6% of the city’s population identified as Black or Brown [24], 11.2% of the population lived in moderate or extreme poverty [25], and in 2018 61.3% of the population received health care through federally-run Community Health Centers (CHCs) [26].

### Study design

This secondary data analysis is based on cross-sectional survey data examining household food insecurity and other factors associated with ECD. The data were collected from CHCs in Brasília, Brazil, between March 2017 and March 2018 [27]. The study protocol was approved by the Human Research Ethics Committee of the Faculty of Health Sciences of the University of Brasília and Health Sciences Teaching and Research Foundation (FEPECS) Ethics Committee (protocol 1.178.564). Participation in the study was voluntary and written informed consent was obtained from all mothers for themselves and on behalf of their participating child.

### Sampling

A two-stage representative sampling approach was used. In the first stage, 20 of the 131 CHCs that monitor child growth and development in Brasília were randomly selected for participation [27]. In the second stage, the number of children to be included from each of the 20 CHCs was estimated based on self-weighted sampling stratified into two age groups (0–12 and 12–24 months). Sample size estimates found that, in order to examine household food insecurity and other ECD-related factors, a minimum sample size of 856 mother-child dyads was required to have a representative sample of children aged 0–24 months who attended the CHCs. This estimate assumed a 95% confidence interval, a 5% error, and allowed for up to 10% losses to follow up.

**Fig 1:**
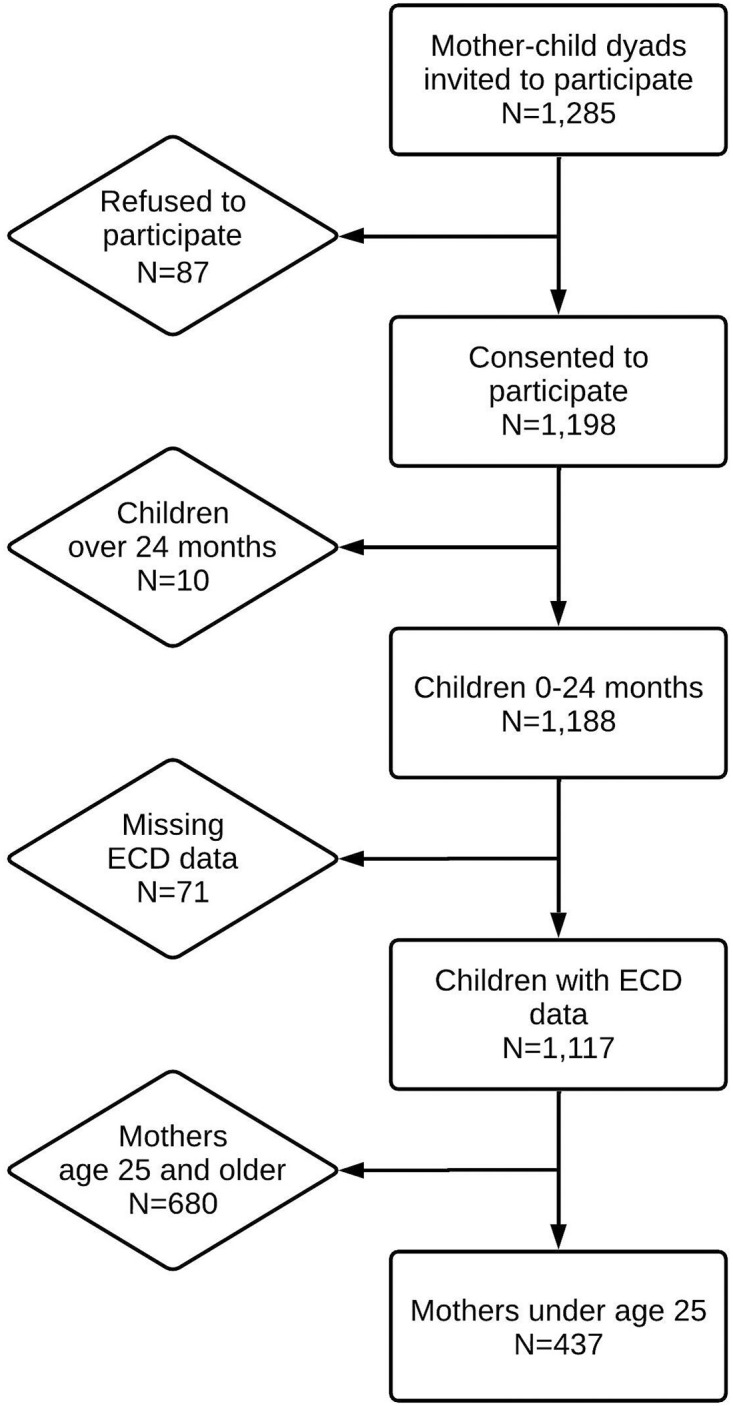
Analytical sample flow chart.

Eligible children included those who were full-term, up to 2 years old, and accompanied by their biological mothers. Children were ineligible if they were born preterm, were twins, had congenital malformations or diagnosed pathologies impacting physical or cognitive development, had undergone major surgery, or had a previous medical diagnosis of developmental delays. Of the total 1,285 mothers invited to participate, 87 (6.77%) refused participation and 81 (6.3%) were excluded, resulting in 1,177 dyads eligible for participation. Details about the final sample and reasons for exclusion are described in Fig 1.

### Analytical sample

The analytical sample for this study was limited to young mothers under 25 years old (Fig 1). For the analysis, we considered three analytical age groups: the full sample of young mothers under 25 years old (N = 437 mother-child dyads), adolescent mothers 13–19 years old (N = 146 dyads), and young adult mothers 20–24 years old (N = 291 dyads). These groupings were determined according to research on adolescent brain development and widely recognized age categorizations, as well as evidence that adolescent mothers have greater health risks compared to young adult mothers, and their infants face higher risks of low birthweight, infection, preterm delivery, and neonatal disability [2, 8]. This analytical sample was used to explore associations between maternal socio-demographic factors and risk of ECD delay for children of adolescent and young adult mothers.

### Data collection

Eligible mothers were administered a survey with close-ended questions related to the child and mother’s demographic, socioeconomic, and biomedical profiles. Standard tools adapted for use with a Brazilian population were used to assess household food insecurity [27]. Quality control was conducted with a random 20% of participants who were contacted by telephone to answer three random survey questions within 4 weeks of participation.

## Measurements

### Outcome

ECD was measured with the Denver Developmental Screening Test II (DDSTII), a screening tool developed to assess a child’s potential developmental risks relative to their age group across four functional domains: personal-social, fine motor, gross motor, and language [28]. This tool had been previously translated and adapted for use in Brazil [29]. According to DDSTII design, children’s assessments corresponded with their age based on categorizations from the American Academy of Pediatrics periodicity schedule [28]. Age-appropriate skills relevant to each domain, such as rolling over, imitating speech, and waving, were evaluated by a trained researcher. The DDSTII classifies developmental skills as normal (0 items performed as delay for age and ≤1 item performed as caution for age) or suspect (≥1 item performed as delay for age and/or ≥2 items performed as caution for age) [28]. The primary outcome variable of this study is risk of ECD delay (no/yes), referring to those children with suspect performance across one or more domains.

ECD was evaluated in a private room in the selected CHC by researchers trained in the DDSTII. To ensure accuracy when applying the test, the researchers answered a DDSTII self-administration checklist during the first ten evaluations. Interobserver reliability analysis was performed in a random subsample by confirming agreement among researchers on their classification of developmental skills across their first ten evaluations (kappa = 0.62, p< 0.0001). After the assessment, mothers of children found to be at risk of ECD delay were provided with information and resources for supporting nurturing care, including adequate child development stimulation, caregiver-bond strengthening, and healthy eating practices. Mothers and children met with the pediatrician immediately following the test administration and mothers were encouraged to discuss the assessment results with the child’s pediatrician in any follow-up appointments.

### Independent variables

Maternal socio-demographic factors were considered the independent variables and included years of education (≥9/<9), employment outside the home (no [including on maternity leave]/yes), mother head of household (no [husband or partner, both husband and wife, or other]/yes), multiparity (no/yes), race (non-Black [white, brown, other]/ Black), and partnership (yes/no [single, divorced, or widowed]). Multiparity was included as a maternal variable due to the percentage of the sample’s young adult mothers who were multiparous and therefore likely to have been adolescent mothers at the time of having their first child.

### Covariates

**Fig 2:**
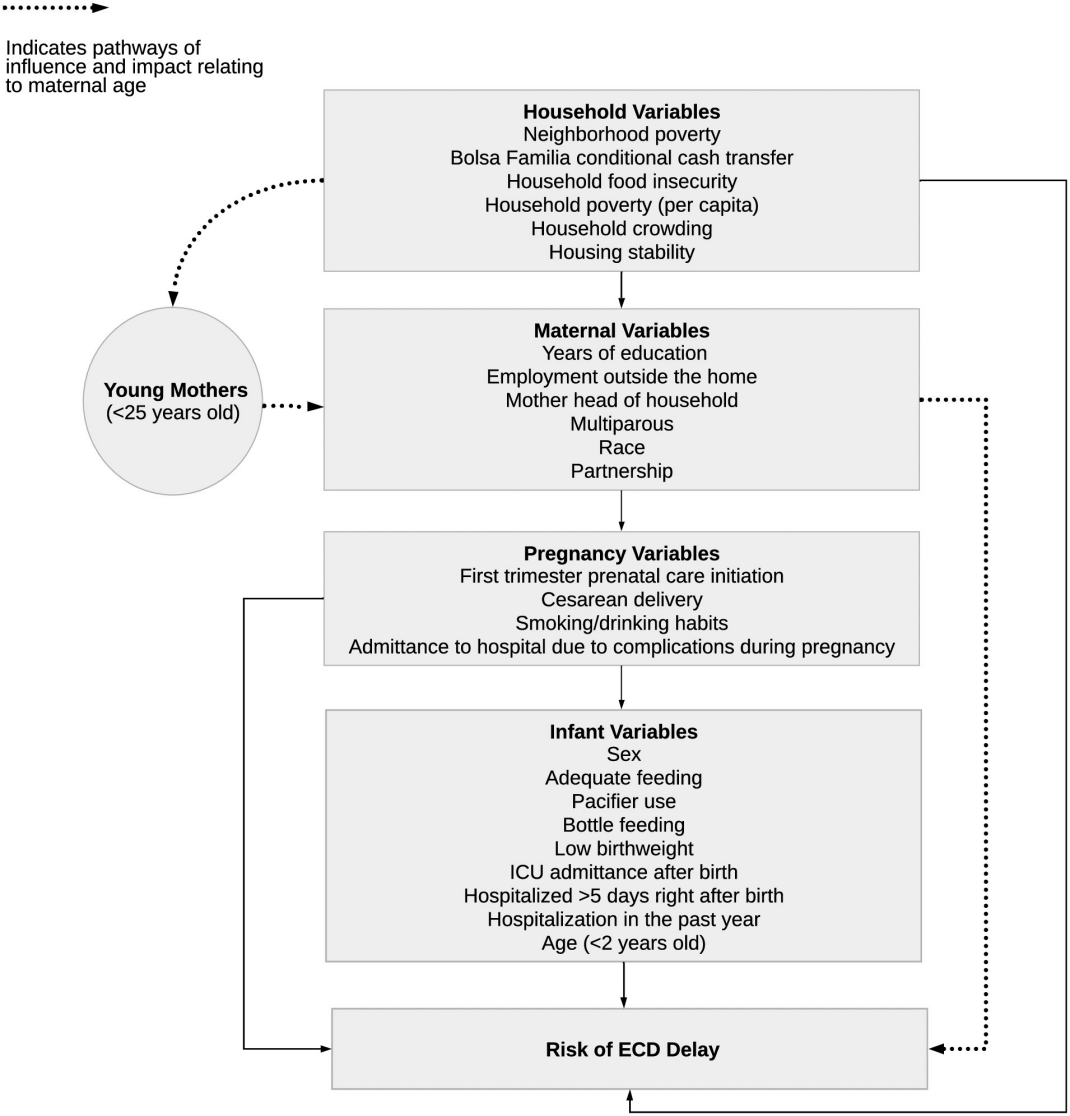
Conceptual model of variables associated with maternal age and ECD outcomes.

Covariate selection was guided by the social-ecological model [30] and empirical evidence [7, 10, 31, 32] supporting associations with both maternal age and ECD outcomes. These determinations informed the conceptual model illustrating potential confounders organized across four categories of variables: household, maternal (independent variables), pregnancy, and infant (Fig 2).

The household variables included neighborhood socio-economic status (medium-high income/medium-low income/low-income), with cut-off points based on the classifications used in the 2018 Brasília household survey [24]. Other variables included Bolsa Familia Conditional Cash Transfer Program recipient (yes/no), household food insecurity (no/yes), household crowding (no/yes [≥2 people over the age of 5 per bedroom]), and housing stability (yes [ownership or paying monthly]/no). Household poverty per capita was defined based on the number of monthly minimum-wage salaries per family; families with 1–3 monthly salaries were classified as ‘poor’ (approximately R$937–2,811 or USD $251–754 per month), while those with 3–4+ monthly salaries were classified as ‘not poor’ (approximately R$2,811–3,748 or USD$754–1,005 per month). Currency conversions from R$ to USD were based on 2018 data from the World Bank [33].

The pregnancy variables included first trimester prenatal care initiation (yes/no), cesarean delivery (no/yes), smoking and drinking habits during pregnancy (neither/smoked and/or drank), and admittance to hospital due to complications during pregnancy (no/yes).

The infant variables included sex (male/female), pacifier use (no/yes), bottle feeding (no/yes), low birthweight (no/yes [≥ 2,500g]), ICU admittance after birth (no/yes), hospitalization for 5 or more days right after birth (no/yes), hospitalization in the past year (no/yes), and age (<6 months/6-24 months old). We also examined adequate feeding (yes/no), defined as 6-month exclusive breastfeeding followed by food diversity; minimum dietary diversity entails consumption of at least 4 food groups, as determined by Bortolini et al.’s adaptation of the World Health Organization indicator for dietary diversity [34, 35].

### Data analysis

Statistical analyses were conducted using STATA MP16. Descriptive analyses of the outcome, independent variables, and covariates were conducted across the full sample and sub-groups of adolescent and young adult mothers. A p-value <0.05 was used as the criterion for statistical significance. Due to sample size limitations, bivariate analyses were only conducted across the full sample of young mother-child dyads to examine the associations between risk of ECD delay, independent variables, and covariates. Following a theory modelling approach [36], variables were selected based on our conceptual model (Fig 2) and only covariates associated with a p-value <0.20 in the bivariate analysis were included and retained in the multivariate model. Multivariate logistic regressions with robust variance were performed for the full sample to estimate variables’ adjusted odds ratios (AORs) and corresponding 95% confidence intervals (CIs), describing the association between risk of ECD delay and maternal variables, and adjusting for covariates.

## Results

The mean age for mothers in the sample was 20.62 years, with a standard deviation of 2.48 years; children in the sample had a mean age of 9.03 months with a standard deviation of 6.40 months. Risk of ECD delay was found in 17.39% (N = 76) of children. Across the full sample of young mothers, 60.36% (N = 236) were living in poverty, 73.17% (N = 319) had ≥9 years of education, and 86.14% (N = 373) were not working outside the home at the time of data collection. Furthermore, 90.11% (N = 392) did not identify as head of their household, and 73.68% (N = 322) were primiparous.

No significant differences were found between children of adolescent mothers and children of young adult mothers regarding their risk of ECD delay (Table 1). However, compared to adolescent mothers, young adult mothers were more likely to have ≥9 years of education (83.16% vs. 53.10%), be employed outside the home (17.07% vs. 7.53%), be head of their household (13.10% vs. 3.45%), and be multiparous (34.36% vs. 10.27%). More adolescent mothers lived in crowded households (15.75% vs. 7.56%) and were unpartnered (54.79% vs. 37.93%) compared to young adult mothers, while fewer adolescent mothers had cesarean birth compared to young adult mothers (23.97% vs. 34.14%). Compared to infants of young adult mothers, more infants of adolescent mothers used pacifiers (63.01% vs. 51.55%) and were bottle-fed (76.03% vs. 67.35%).

**Table 1:** Characteristics of adolescent and young adult mothers with children under 2 years old by maternal age, Brasília, Brazil, 2017–18.

Variables	Full Sample (N = 437)	Adolescent Mothers (N = 146)	Young Adult Mothers (N = 291)	p
	% (N*)	% (N*)	% (N*)	
**ECD outcome**				
Suspect ECD	17.39 (76)	17.12 (25)	17.53 (51)	0.917
**Household variables**				
Socio-economic status				0.375
Medium-high income	21.97 (96)	19.18 (28)	23.37 (68)	
Medium-low income	53.55 (234)	52.74 (77)	53.95 (157)	
Low-income	24.49 (107)	28.08 (41)	22.68 (66)	
Bolsa Familia conditional cash transfer recipient				0.247
Yes	18.08 (79)	15.07 (22)	19.59 (57)	
No	81.92 (358)	84.93 (124)	80.41 (234)	
Household food insecurity				0.712
No	62.33 (268)	61.11 (88)	62.94 (180)	
Yes	37.67 (162)	38.98 (56)	37.06 (106)	
Household poverty (per capita)				0.065
No poverty	39.64 (155)	33.07 (42)	42.80 (113)	
Poverty	60.36 (236)	66.93 (85)	57.20 (151)	
Household crowding				0.008
Not crowded	89.70 (392)	84.25 (123)	92.44 (269)	
Crowded	10.30 (45)	15.75 (23)	7.56 (22)	
Housing stability				0.628
Yes	81.61 (355)	82.88 (121)	80.97 (234)	
No	18.39 (80)	17.12 (25)	19.03 (55)	
**Maternal variables**				
Years of education				<0.001
≥9	73.17 (319)	53.10 (77)	83.16 (242)	
<9	26.83 (117)	46.90 (68)	16.84 (49)	
Employment outside the home				0.007
No	86.14 (373)	92.47 (135)	82.93 (238)	
Yes	13.86 (60)	7.53 (11)	17.07 (49)	
Mother head of household				0.001
No	90.11 (392)	96.55 (140)	86.90 (252)	
Yes	9.89 (43)	3.45 (5)	13.10 (38)	
Multiparous				<0.001
No	73.68 (322)	89.73 (131)	65.64 (191)	
Yes	26.32 (115)	10.27 (15)	34.36 (100)	
Race				0.107
Non-Black	83.49 (364)	79.45 (116)	85.52 (248)	
Black	16.51 (72)	20.55 (30)	14.48 (42)	
Partnership				0.001
Yes	56.42 (246)	45.21 (66)	62.07 (180)	
No	43.58 (190)	54.79 (80)	37.93 (110)	
**Pregnancy variables**				
First trimester prenatal care initiation				0.227
Yes	77.88 (338)	74.48 (108)	79.58 (230)	
No	22.12 (96)	25.52 (37)	20.42 (59)	
Cesarean delivery				0.030
No	69.27 (302)	76.03 (111)	65.86 (191)	
Yes	30.73 (134)	23.97 (35)	34.14 (99)	
Smoking/drinking habits				0.128
No	85.78 (374)	82.19 (120)	87.59 (254)	
Yes	14.22 (62)	17.81 (26)	12.41 (36)	
Admittance to hospital due to complications during pregnancy				0.868
No	83.30 (364)	82.88 (121)	83.51 (243)	
Yes	16.70 (73)	17.12 (25)	16.49 (48)	
**Infant variables**				
Sex				0.974
Male	48.05 (210)	47.95 (70)	48.11 (140)	
Female	51.95 (227)	52.05 (76)	51.89 (151)	
Adequate feeding				0.226
Yes	59.50 (260)	55.48 (81)	61.51 (179)	
No	40.50 (177)	44.52 (65)	38.49 (112)	
Pacifier use				0.023
No	44.62 (195)	36.99 (54)	48.45 (141)	
Yes	55.38 (242)	63.01 (92)	51.55 (150)	
Bottle feeding				0.061
No	29.75 (130)	23.97 (35)	32.65 (95)	
Yes	70.25 (307)	76.03 (111)	67.35 (196)	
Low birthweight				0.132
No	94.39 (387)	91.97 (126)	95.60 (261)	
Yes	5.61 (23)	8.03 (11)	4.40 (12)	
ICU admittance after birth				0.454
No	96.78 (421)	95.89 (140)	97.23 (281)	
Yes	3.22 (14)	4.11 (6)	2.77 (8)	
Hospitalization for 5 or more days right after birth				0.104
No	83.52 (365)	79.45 (116)	85.57 (249)	
Yes	16.48 (72)	20.55 (30)	14.43 (42)	
Hospitalization in the past year				0.932
No	89.22 (389)	89.04 (130)	89.31 (259)	
Yes	10.78 (47)	10.96 (16)	10.69 (31)	
Age				0.807
<6 months old	42.33 (185)	43.15 (63)	41.92 (122)	
6–24 months old	57.67 (252)	56.85 (83)	58.08 (169)	

*Numbers may not sum to total due to missing data.

Bivariate analyses across the full sample indicated that risk of ECD delay was significantly associated with mother’s fewer years of education (OR = 1.69; 95% CI, 1.00–2.87), working outside the home (OR = 1.90; 95% CI, 1.00–3.58), being head of their household (OR = 2.26; 95% CI, 1.12–4.58), multiparity (OR = 2.27; 95% CI, 1.35–3.82), and household food insecurity (OR = 1.73; 95% CI, 1.04–2.87) (Table 2). Among the infant variables examined, adequate feeding, pacifier use, bottle feeding, low birthweight, and hospitalization for 5 or more days right after birth were found to be associated with an increased risk of ECD delay.

**Table 2:** Unadjusted odds ratio (OR) and 95% confidence interval (CI) for risk of ECD delay among children under 2 years old of adolescent and young adult mothers, Brasília, Brazil, 2017–18.

Variables	Full Sample (N = 437)			
	N*	% (N) at risk of ECD delay	OR [95% CI]	p
**Household variables**				
Socio-economic status				
Medium-high income	96	14.58 (14)	1	
Medium-low income	234	15.81 (37)	1.10 [0.56–2.14]	0.779
Low-income	107	23.36 (25)	1.79 [0.87–3.68]	0.116
Bolsa Familia conditional cash transfer recipient				
Yes	79	17.72 (14)	1	
No	358	17.32 (62)	0.97 [0.51–1.84]	0.932
Household food insecurity				
No	268	14.18 (38)	1	
Yes	162	22.22 (36)	1.73 [1.04–2.87]	0.034
Household poverty (per capita)				
No poverty	155	16.77 (26)	1	
Poverty	236	20.76 (49)	1.30 [0.77–2.20]	0.328
Household crowding				
Not crowded	392	17.86 (70)	1	
Crowded	45	13.33 (6)	0.71 [0.29–1.74]	0.450
Housing stability				
Yes	355	18.31 (65)	1	
No	80	13.75 (11)	0.71 [0.36–1.42]	0.334
**Maternal variables**				
Years of education				
≥9 or more	319	15.05 (48)	1	
<9	117	23.08 (27)	1.69 [1.00–2.87]	0.051
Employment outside the house				
No	373	16.09 (60)	1	
Yes	60	26.67 (16)	1.90 [1.00–3.58]	0.048
Mother head of household				
No	392	16.07 (63)	1	
Yes	43	30.32 (13)	2.26 [1.12–4.58]	0.023
Multiparous				
No	322	13.98 (45)	1	
Yes	115	26.96 (31)	2.27 [1.35–3.82]	0.002
Age				
20–24 years old	291	17.53 (51)	1	
13–19 years old	146	17.12 (25)	1.03 [0.61–1.74]	0.917
Race				
Non-Black	364	17.31 (63)	1	
Black	72	18.06 (13)	1.05 [0.54–2.03]	0.879
Partnership				
Yes	246	18.29 (45)	1	
No	190	16.32 (31)	0.87 [0.53–1.44]	0.590
**Pregnancy variables**				
First trimester prenatal care initiation				
Yes	338	17.16 (58)	1	
No	96	17.71 (17)	1.04 [0.57–1.88]	0.900
Cesarean delivery				
No	302	17.22 (52)	1	
Yes	134	17.91 (24)	1.05 [0.62–1.79]	0.861
Smoking/drinking habits				
No	374	17.11 (64)	1	
Yes	62	19.35 (12)	1.16 [0.59–2.31]	0.667
Admittance to hospital due to complications during pregnancy				
No	364	17.86 (65)	1	
Yes	72	15.28 (11)	0.83 [0.41–1.66]	0.599
**Infant variables**				
Sex				
Male	210	13.81 (29)	1	
Female	227	20.70 (47)	1.63 [0.98–2.71]	0.059
Adequate feeding				
Yes	260	13.08 (34)	1	
No	177	23.72 (42)	2.07 [1.25–3.41]	0.004
Pacifier use				
No	195	11.79 (23)	1	
Yes	242	21.90 (53)	2.10 [1.23–3.57]	0.006
Bottle feeding				
No	130	9.23 (12)	1	
Yes	307	20.85 (64)	2.59 [1.35–4.98]	0.004
Low birthweight				
No	387	14.47 (56)	1	
Yes	23	43.48 (10)	4.55 [1.90–10.87]	0.001
ICU admittance after birth				
No	421	17.34 (73)	1	
Yes	14	21.43 (3)	1.30 [0.35–4.78]	0.693
Hospitalization for 5 or more days right after birth				
No	365	15.62 (57)	1	
Yes	72	26.39 (19)	1.94 [1.07–3.51]	0.030
Hospitalization in the past year				
No	389	16.45 (64)	1	
Yes	47	25.53 (12)	1.74 [0.86–3.54]	0.125
Age				
<6 months old	185	14.05 (26)	1	
6–24 months old	252	19.54 (50)	1.51 [0.90–2.54]	0.116

*Numbers may not sum to total due to missing data.

Among the maternal socio-demographic variables, adjusted logistic regression found only multiparity to be an independent factor associated with increased risk of ECD delay across the full sample of young mother-child dyads (AOR = 2.51; 95% CI, 1.23–5.13) (Table 3). Among the infant variables, inadequate feeding (AOR = 2.62; 95% CI, 1.43–4.78), low birthweight (AOR = 4.30; 95% CI, 1.58–11.70), and hospitalization for 5 or more days right after birth (AOR = 2.48; 95% CI, 1.19–5.16) were independently associated with a risk of ECD delay in the full sample.

**Table 3:** Adjusted odds ratio (AOR) and 95% confidence interval (CI) for risk of ECD delay among children under 2 years old of adolescent and young adult mothers, Brasília, Brazil, 2017–18.

Variables	Risk of ECD delay	
	AOR [95% CI]	p
**Household variables**		
Socio-economic status		
Medium-high income	1	
Medium-low income	0.96 [0.43–2.15]	0.914
Low-income	1.84 [0.82–4.13]	0.140
Household food insecurity		
No	1	
Yes	0.92 [0.48–1.78]	0.813
**Maternal variables**		
Years of education		
≥9	1	
<9	1.08 [0.55–2.11]	0.829
Employment outside the house		
No	1	
Yes	1.43 [0.68–3.01]	0.348
Mother head of household		
No	1	
Yes	2.18 [0.91–5.26]	0.081
Multiparous		
No	1	
Yes	2.52 [1.23–5.17]	0.011
**Infant variables**		
Sex		
Male	1	
Female	1.39 [0.75–2.58]	0.298
Adequate feeding		
Yes	1	
No	2.67 [1.44–4.97]	0.002
Pacifier use		
No	1	
Yes	0.91 [0.41–2.02]	0.820
Bottle feeding		
No	1	
Yes	2.20 [0.98–4.97]	0.057
Low birthweight		
No	1	
Yes	4.45 [1.62–12.20]	0.004
Hospitalization for 5 or more days right after birth		
No	1	
Yes	2.52 [1.20–5.31]	0.015
Hospitalization in the past year		
No	1	
Yes	0.68 [0.22–2.06]	0.496
Age		
<6 months old	1	
6–24 months old	1.30 [0.69–2.44]	0.413

## Discussion

In this study, multiparity was the only independent maternal factor found to be associated with an increased risk of ECD delay for children of young mothers in Brazil. While the association between high parity and suspected developmental delays in children at 12 months in Brazil has been previously identified [37], less is known about the associations between multiparity of adolescent and young adult mothers and ECD outcomes. Maternal multiparity in non-adolescent mothers has been identified as a postpartum stressor impacting maternal physical and mental health; the complexities of managing time and resources to care for multiple children often lead mothers to continually prioritize their children’s needs over their own [38–41]. Multiparity has also been associated with higher rates of social vulnerability, which itself is a risk factor for increased household food insecurity [42]. High parity, poor caregiver mental health, and malnutrition have all been identified as threats to ECD [7, 37, 43]. Our findings on young adult and adolescent mothers in Brazil corroborate the challenges to ECD described in existing literature, although few of those studies focused on adolescent women.

The impacts of early pregnancy have been well-documented as compromising young parents’ current and future health, as well as the health of their children [2, 3, 44]. Pregnancy in young mothers has been associated with heightened risk of poverty and limited opportunities for education and employment [3, 37]. Compared to their non-adolescent counterparts, adolescent mothers and their infants face higher rates of pregnancy-related adverse health outcomes [9, 44, 45], higher rates of unmet need for modern contraception [46], and increased likelihood of unsafe abortion and resulting maternal morbidity and mortality [2, 39]. Globally, complications from pregnancy and childbirth are the leading cause of death for adolescent girls age 15–19. These risks are compounded for young adult and adolescent mothers when early pregnancy is experienced in conjunction with a loss of opportunity for continued education, skill development, and engagement with supportive social networks [3, 47]. In Brazil, adolescent childbirth and multiparity have been associated with worse physical performance later in life for low-income women [48]. Other factors found to be associated with adolescent multiparity include intention to have a first pregnancy, a previous poor obstetrical outcome, and having a partner with an intention for a repeat pregnancy [49].

Although not independently significant in this sample, other maternal socio-demographic factors such as fewer years of education, lack of employment outside the home, and mother-headed households increased the risk of ECD delay. In Brazil, more years of education have been associated with higher rates of contraception use and lower chances of early pregnancy across all classes [6, 50], while pregnancy in adolescence has been associated with lower rates of school completion and persistent economic and social inequities [19]. Furthermore, lack of child care has been reported as a primary barrier to regular school attendance or school completion for young parents [51], highlighting the intersectional nature of the socio-demographic factors impacting young parent’s capacity to provide nurturing care that promotes ECD. In addition, young people can face significant barriers to accessing health care, including a relative lack of knowledge and experience, prohibitive out-of-pocket costs, and restrictive policies that perpetuate stigma and make it harder to receive comprehensive, confidential services [32]. While sexual and reproductive health education is an integral aspect of adolescent health care [52], underlying socio-economic factors have been more clearly associated with changes in adolescent fertility rates [53]. Wide-ranging evidence links educational attainment to future employment, health, and wellbeing which in turn supports parents in providing nurturing-care environments that promote favorable patterns of development and help buffer against biological and environmental threats to ECD [9, 32, 39, 54]. Therefore, multisectoral policies and interventions that connect adolescent health, parenting educational programming, and ECD should be further explored in Brazil to minimize structural inequities and ensure that young parents are able to protect their own health and that of their children.

Extensive evidence documents the connections between low birthweight, inadequate feeding, early hospitalization, and suboptimal ECD [55–57], supporting our study’s findings that infant variables (aside from child age and sex) were factors independently associated with risk of ECD delay. Previous studies have identified several factors that go beyond low socioeconomic status to contextualize the multiple determinants of low birthweight in children of adolescent mothers, including: biological immaturity, inadequate prenatal care, inadequate weight gain during pregnancy, and unhealthy behavior during pregnancy [2, 58]. A cross-sectional study of 4,746 mother-infant dyads in Brazil found that adolescent pregnancy increased the risk for low birthweight only for mothers without partners [59].

Positive paternal involvement has been found to support breastfeeding, maternal mental health, and infant development [60–64], while greater social support and family functioning has been associated with improved parenting behaviors, satisfaction, and self-efficacy [65, 66]. As such, future studies are needed to investigate the effects of paternal involvement as a means of improving ECD by increasing social support for maternal headed households and mothers working outside the home.

Regarding infant feeding practices, consistent evidence has documented lower breastfeeding duration among adolescent mothers [67] due to a lack of knowledge of breastfeeding benefits, obstacles in sharing caregiving time between children, experiences of stigma from society and peers, going back to school, and social activities with friends [68].

The infant variables explored in this sample are related to maternal health and social factors given that childhood nutritional deficiencies often co-occur with extreme poverty, food insecurity, less responsive parenting, and exposure to domestic violence or pathogens [4, 5, 10, 69]. Accordingly, there is a need to develop interventions that improve family income, parenting skills, and material resources from preconception through adolescence to support nurturing care and positive ECD [9].

### Strengths and limitations

Our study is limited by the small sample size of adolescent and young adult mothers, preventing analytical exploration by maternal age sub-group as originally planned. While the sample size may have resulted in select variables losing statistical significance in the multivariate model, we followed an evidence-based theory modelling approach which acknowledges that some real, explanatory variables with causal effects on the dependent variable may not be statistically significant [36]. Therefore, we believe the selection of co-variables, as guided by the evidence-based explanatory conceptual model (Fig 2), builds a strong case for the findings presented.

Since our sample included parents that receive services through federally-run CHCs targeting families with low incomes, our study may have overestimated the prevalence of risk of ECD given the lower rates of prenatal care among those using the national health care system [58]. Additionally, we did not screen for children with a previous diagnosis of a developmental delay or any major medical conditions or surgery, which could overestimate prevalence of ECD delays. Importantly, the DDSTII is not meant to provide a diagnosis but rather is intended as a screening tool to measure ECD, the outcomes of which must be interpreted in the context of the child’s wider nurturing care environment [28]. We acknowledge that the study’s cross-sectional design does not clarify temporal relationships among determinants or ECD outcomes; however, cross-sectional studies are useful to raise hypotheses for future studies [70] regarding the specific policies and programmatic components most effective at promoting adolescent and young adult maternal health to support optimal ECD.

Our study sample from Brasília is representative of certain pregnancy trends across the country. It was found in São Paulo that more adolescent mothers receive services through the national health system than through private care; these adolescents were found to have lower rates of prenatal care and instruction, and higher rates of multiparty and cesarean than their counterparts using the private health system [71]. Given Brasília’s socio-economic similarities to other large Brazilian cities, and the nationally and internationally-representative rates of adolescent fertility, our study’s focus on adolescent pregnancy can provide insight for local, national, and international contexts. Therefore, our results add to the body of evidence indicating multiparity as a factor for increased risk of ECD delay, expanding on previous studies to broaden this association to include the children under 2 years old of adolescent and young adult mothers in Brazil.

### Conclusion

In addition to other well-known factors influencing ECD delay, our study found multiparity to be independently associated with an increased frequency of risk of ECD delay among children under age 2 of adolescent and young adult mothers in Brazil. Our findings indicate the influence of socio-demographic factors on adolescent and young adult mothers’ capacity to support ECD-nurturing environments for their children, underscoring the need for multisectoral policies and parenting interventions for young primiparous and multiparous mothers. Further investigation is needed to understand how initiatives that connect family-based health interventions with ECD programming can improve health outcomes for adolescents, women, children, and families throughout Brazil.
